# Unraveling the oral microbiome's role in Alzheimer's disease: From pathophysiology to therapeutic potential

**DOI:** 10.1002/alz.71011

**Published:** 2025-12-13

**Authors:** Gilliana Rozenblum, Karima Ait‐Aissa, Gadeer Zahran, Mahdieh Alipour, Amal M. Sahyoun, Undral Munkhsaikhan, Adam Kassan, Tauheed Ishrat, Qi Wang, Ammaar H. Abidi, Modar Kassan

**Affiliations:** ^1^ College of Dental Medicine Lincoln Memorial University Knoxville Tennessee USA; ^2^ School of Pharmacy West Coast University Los Angeles California USA; ^3^ Department of Anatomy and Neurobiology University of Tennessee Health Science Center Memphis Tennessee USA; ^4^ Department of Pharmaceutical Sciences College of Pharmacy The University of Tennessee Health Science Center Memphis Tennessee USA; ^5^ Neuroscience Institute University of Tennessee Health Science Center Memphis Tennessee USA

**Keywords:** Alzheimer's disease, biomarkers, microbial translocation, neuroinflammation, oral microbiome, periodontal pathogens, systemic inflammation, therapeutic interventions

## Abstract

Oral dysbiosis contributes to Alzheimer's disease (AD) by promoting neuroinflammation. Pathobionts such as *Porphyromonas gingivalis*, *Treponema denticola*, and *Fusobacterium nucleatum* release virulence factors that induce amyloid beta aggregation and tau hyperphosphorylation, while the loss of commensals like *Streptococcus salivarius* and *Neisseria spp*. impairs anti‐inflammatory protection, worsening neuronal damage. *P. gingivalis* is strongly linked to an increased risk of AD, especially in individuals with systemic conditions like diabetes, hypertension, and chronic kidney disease. Its presence in brain tissue correlates with a higher likelihood of AD, while salivary *Veillonella* and periodontal pathogens in gingival crevicular fluid show potential as non‐invasive biomarkers for early AD detection. Therapeutic strategies targeting the oral microbiota, such as gingipain inhibitors, antimicrobials, probiotics, and prebiotics, show promise for mitigating AD risk. However, causal mechanisms and clinical efficacy remain to be fully established. Maintaining microbial balance through preventive and targeted modulation represents an innovative approach to reducing AD susceptibility.

**Highlights:**

- We identified *Porphyromonas gingivalis*, *Treponema denticola*, and *Fusobacterium nucleatum* as key oral pathogens driving Alzheimer's disease (AD) via gingipain‐induced amyloid beta aggregation, systemic inflammation, and blood–brain barrier disruption.
- Our study revealed diabetes, hypertension, and chronic kidney disease (CKD) amplify AD risk through shared oral dysbiosis, with uremic toxins (CKD) and hyperglycemia (diabetes) exacerbating neuroinflammation.
- We propose *Veillonella* in saliva and *Porphyromonas gingivalis* in gingival crevicular fluid as non‐invasive AD biomarkers, correlating with 6 to 10× higher AD risk when detected in brain tissue.
- Gingipain inhibitors (e.g., COR388), nitrate‐reducing probiotics, and integrated dental‐neurology care are promising interventions to disrupt the oral–brain axis.
- We advocate for oral microbiome screening in high‐risk populations (apolipoprotein E ε4 carriers, diabetics) and interdisciplinary approaches to AD prevention.

## 1 INTRODUCTION

Alzheimer's disease (AD) represents one of the most challenging neurodegenerative disorders of our time, marked by progressive cognitive decline and memory impairment.^1^ AD is increasingly understood as a multifactorial disorder, with hypotheses encompassing amyloid beta (Aβ) accumulation, tau pathology, neuroinflammation, vascular dysfunction, and infectious agents.^2^ Microorganisms may access the central nervous system (CNS) through multiple routes, including hematogenous dissemination and neuroanatomical pathways such as the trigeminal, olfactory, and vagus nerves. While the accumulation of Aβ plaques and neurofibrillary tangles remains central to its pathology, emerging research highlights the potential influence of systemic factors, including microbial dysbiosis, in disease progression.^1^ Among these, the oral microbiota has garnered increasing attention as a possible contributor to AD pathogenesis through both direct and indirect mechanisms.

The oral cavity harbors a complex ecosystem of microorganisms that play critical roles in maintaining oral and systemic health.^3^ Under normal conditions, this microbial community exists in a state of equilibrium, contributing to immune regulation and metabolic homeostasis.^4^ However, disruptions in this balance, often driven by poor oral hygiene, chronic inflammation, or systemic disease, can lead to dysbiosis and the proliferation of pathobiont species such as *Porphyromonas gingivalis*, *Treponema denticola*, and *Fusobacterium nucleatum*.^5^ These organisms produce virulence factors. For example*, P. gingivalis* produces gingipains, which are cysteine proteases, as well as lipopolysaccharides (LPS) that disrupt host immune and compromise endothelial and epithelial barrier integrity; *T. denticola* expresses dentilisin and lipooligosaccharide (LOS), which activate Toll‐like receptor‐mediated inflammatory cascades, while *F. nucleatum* uses adhesins such as FadA and Fap2 to promote host cell invasion and cytokine release. These virulence factors not only exacerbate periodontal disease but also have the potential to disseminate systemically, crossing the blood–brain barrier (BBB) resulting in an increase in its permeability, and contributing to neuroinflammation.^6^

Growing evidence supports a bidirectional relationship between oral microbial dysbiosis and AD pathology, suggesting a reinforcing cycle of neuroinflammation and microbial imbalance.^7^ Although *P. gingivalis* DNA and gingipains have been detected in AD brains, some evidence suggests that Aβ aggregation may represent a host defense mechanism against microbial invasion rather than purely pathogenic deposition.^8^, ^9^ Furthermore, chronic periodontitis has been associated with accelerated cognitive decline, with studies demonstrating that individuals with severe periodontal disease exhibit a significantly higher risk of developing mild cognitive impairment and AD.^10^ These observations are supported by preclinical models, in which oral pathobiont inoculation has been shown to induce neuroinflammation, neuronal damage, and amyloid deposition.

Beyond direct microbial invasion, oral dysbiosis may also exacerbate AD progression through systemic inflammation.^10^ Periodontal pathogens can trigger the release of pro‐inflammatory cytokines, which may compromise BBB integrity and activate microglial cells, perpetuating a cycle of neuroinflammation and neurodegeneration.^11^ Additionally, the oral microbiota's interaction with the gut–brain axis presents another plausible pathway, as oral pathogens can alter gut microbiota composition, further amplifying systemic inflammatory responses that may impact central nervous system health.^12^ Neuroinflammation represents a central link between peripheral dysbiosis and AD pathology. Periodontal pathogens trigger microglial activation via Toll‐like receptor (TLR)2/4 pathways, upregulating interleukin (IL)‐1β, IL‐6, and tumor necrosis factor alpha (TNF‐α). Chronic cytokine release perpetuates astrocyte activation, synaptic pruning, and neuronal apoptosis, forming a feed‐forward cycle of neurodegeneration.

Despite these compelling associations, critical questions remain regarding the causality and specificity of the oral microbiota's role in AD.^7^ Current studies are largely observational or rely on animal models, leaving gaps in our understanding of the precise mechanisms linking oral dysbiosis to neurodegeneration.^13^ This review synthesizes existing evidence to evaluate the hypothesis that oral microbiota dysbiosis contributes to AD development and progression. By examining clinical, epidemiological, and mechanistic data, we aim to clarify the potential of oral microbiota modulation as a novel strategy for AD prevention and therapy, while identifying key areas for future research to establish definitive causal relationships. A comprehensive description of the literature search and study selection methodology is available in the Supporting Information.

## 2 THE ORAL MICROBIOTA: COMPOSITION, DYNAMICS, AND CLINICAL SIGNIFICANCE

The oral cavity represents a complex and dynamic ecosystem that hosts the second most diverse microbial community in the human body, surpassed only by the gastrointestinal tract.^14^ Characterized by stable temperature, high humidity, and abundant nutrients from food residues and epithelial debris, this environment supports a remarkably varied microbiota that colonizes distinct habitats, including the teeth, tongue, gingiva, and mucosal surfaces.^15^ Microbial colonization begins with bacterial adhesion to salivary pellicles, followed by biofilm formation through cell division and extracellular matrix production.^14^ In a healthy state, these biofilms maintain a symbiotic relationship with the host, dominated by commensal species such as *Streptococcus*, *Veillonella*, and *Actinomyces*.^16^ However, ecological disturbances caused by dietary changes, poor hygiene, or host factors can disrupt this balance, leading to dysbiosis and the emergence of pathogenic communities.^17^

Shifts in microbial composition and function mark the transition from health to disease.^17^ Dental caries, for instance, involve acid‐producing bacteria like *Lactobacillus* and *Streptococcus mutans*, which metabolize dietary sugars to create demineralizing acidic conditions.^18^ Periodontal diseases, including gingivitis and periodontitis, are associated with anaerobic Gram‐negative bacteria such as *P*. *gingivalis*, *Tannerella forsythia*, and *T. denticola*.^19^ These pathogens trigger chronic inflammation, tissue destruction, and systemic immune responses.^20^ Advances in sequencing technologies have expanded our understanding of oral microbial diversity, revealing ≈ 834 prokaryotic species alongside fungi, archaea, and viruses^21^ (https://forsyth.org/ada‐forsyth‐announces‐version‐4‐update‐to‐human‐oral‐microbiome‐database‐including‐expanded‐whole‐genome‐sequence‐information/). Notably, the Human Oral Microbiome Database (HOMD) has cataloged and made these species publicly available online, highlighting dominant phyla including *Firmicutes*, *Bacteroidetes*, and *Proteobacteria*.^22^

Despite considerable interpersonal variation, a core oral microbiota exists, consisting of taxa consistently present across healthy individuals.^14^ This core community contributes to microbial stability and host defense, though its composition varies across oral niches.^23^ For example, the dorsal tongue harbors anaerobic genera like *Prevotella* and *Fusobacterium*, while supragingival plaque is enriched with *Streptococcus* and *Corynebacterium*.^24^ Saliva, lacking a native microbiota, instead reflects microbial shedding from oral surfaces.^24^ The resilience of this ecosystem is influenced by factors ranging from diet and hygiene to systemic health, with dysbiosis implicated not only in oral diseases but also in systemic conditions, including neurodegenerative disorders.^25^ Understanding the intricate balance of the oral microbiota thus holds significant implications for both oral and systemic disease prevention and management.

## 3 CHANGES IN ORAL BACTERIA AND THEIR LINK TO AD

The oral microbiota plays a crucial role in systemic health, and its dysregulation has been increasingly linked to neurodegenerative diseases, particularly AD.^26^ Emerging evidence suggests that AD is associated with a distinct shift in oral microbial composition, characterized by the proliferation of pathogenic bacteria and a decline in beneficial commensal species.^27^ These alterations may contribute to neuroinflammation, Aβ accumulation, and cognitive decline through direct and indirect mechanisms.^28^

Several studies have identified a significant enrichment of periodontal pathogens in the oral and brain tissues of AD patients.^29^ *P. gingivalis*, a keystone periodontitis pathogen, has been consistently found in higher abundance in AD patients, including within *post mortem* brain tissue.^30^ This bacterium produces gingipains, proteases that promote Aβ accumulation and neuroinflammation in animal models, suggesting a potential mechanistic link to AD pathogenesis.^31^ Similarly, *T. denticola*, another periodontitis‐associated bacterium, exhibits increased prevalence in AD patients and has been shown to induce neuronal apoptosis and Aβ deposition in experimental studies.^13^, ^32^

Other pro‐inflammatory species, such as *Aggregatibacter actinomycetemcomitans*, contribute to AD‐related pathology through their LPSs, which trigger systemic inflammation and Aβ secretion in hippocampal neurons.^33^ Additionally, *F. nucleatum* and *Actinomyces meyeri* have been found in elevated proportions in the oral and brain tissues of AD patients, with *A. meyeri* inoculation in mice leading to increased cortical Aβ plaques and cognitive deficits.^34^, ^35^ It is important to mention that bacterial DNA from oral pathobionts has been detected in brain tissues of AD patients and in animal models. However, the presence of a resident or viable brain microbiota remains unproven.

In contrast to the overgrowth of pathogenic species, certain beneficial oral bacteria appear to be reduced in AD patients. *Streptococcus salivarius*, a commensal species known for its protective role against pathogenic overgrowth, is significantly diminished in AD, potentially exacerbating dysbiosis.^36^ Similarly, *Neisseria* species, which are generally non‐pathogenic and contribute to microbial diversity, show lower abundance in AD patients compared to healthy controls.^37^ This depletion of commensal bacteria may disrupt oral microbial homeostasis, facilitating the dominance of pro‐inflammatory pathogens and amplifying systemic inflammation linked to neurodegeneration.

The shift toward a pathogenic oral microbiota in AD may contribute to disease progression through multiple pathways. Periodontal pathogens can enter systemic circulation via bacteremia or through peripheral immune activation, leading to chronic low‐grade inflammation.^38^ Bacterial byproducts such as LPS and gingipains may cross the BBB, directly promoting neuroinflammation and Aβ deposition.^39^ Furthermore, the loss of protective commensals may impair mucosal immunity, allowing pathogenic species to thrive and perpetuate systemic inflammation.

A recent systematic review by Pruntel et al.^40^ analyzed 13 studies comparing oral microbiomes between AD patients and controls, reinforcing these observations. The review identified consistent increases in *P. gingivalis*, *Prevotella intermedia*, and *F. nucleatum* in AD, alongside decreases in health‐associated species such as *Rothia dentocariosa* and *Haemophilus parainfluenzae*.^41^, ^42^ However, the review also highlighted heterogeneity in microbial diversity assessments, with some studies reporting decreased alpha diversity in AD,^43^ while others found no significant changes^44^ or even increased diversity.^42^

Recent investigations have explored the diagnostic potential of oral microbiome profiling in AD^45^ and found that *Veillonella parvula* was significantly enriched in AD patients across saliva and gingival crevicular fluid (GCF) samples, while *P. gingivalis* was specifically elevated in GCF. These microbial signatures correlated with disease severity, suggesting their utility as non‐invasive biomarkers. Similarly, Adnan et al.^46^ reported that cognitively impaired individuals exhibited higher abundances of periodontitis‐associated genera (*Treponema, Parvimonas*) and reduced levels of *Gemella*, a genus potentially protective against cognitive decline.

A meta‐analysis^37^ further strengthened these associations, demonstrating that the presence of oral bacteria in brain tissue, particularly *P. gingivalis*, was linked to a 6‐fold to 10‐fold increased AD risk. These findings underscore the potential role of oral microbiota dysbiosis in AD pathogenesis and highlight the need for longitudinal studies to determine causality.

**FIGURE 1:**
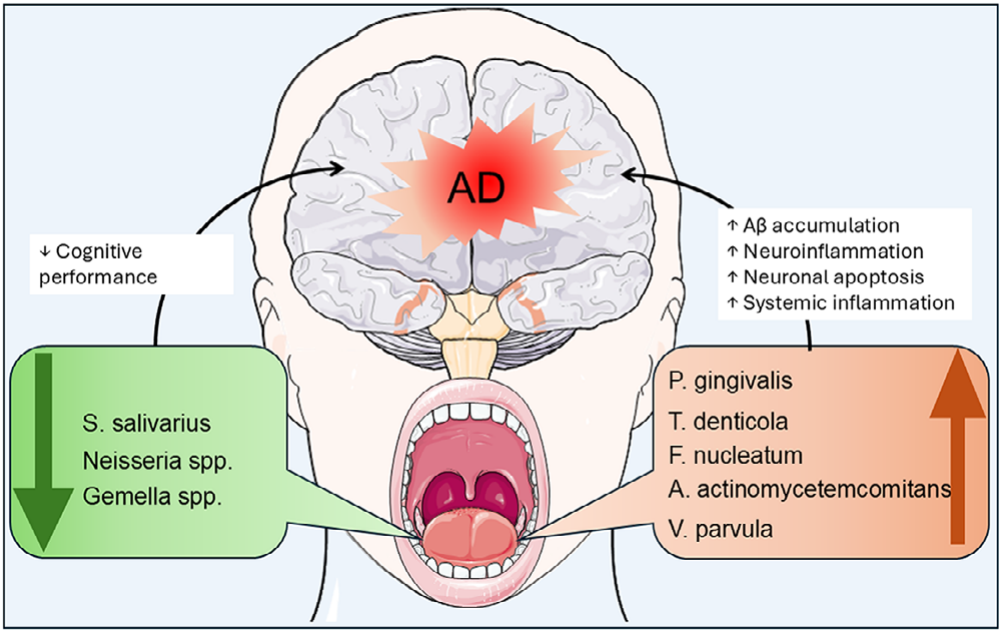
Oral microbial dysbiosis in Alzheimer's disease (AD) showing pathogenic enrichment (*Porphyromonas gingivalis, Treponema denticola, Aggregatibacter actinomycetemcomitans, Fusobacterium nucleatum, Actinomyces meyeri*) and commensal depletion (*Streptococcus salivarius, Neisseria spp., Gemella spp*.), with proposed mechanisms including systemic and neuroinflammation, amyloid beta (Aβ) accumulation, and neuronal apoptosis

In AD and mild cognitive impairment, oral microbiome profiles show enrichment of periodontopathogenic genera with concurrent losses of health‐associated commensals, a pattern observed both in saliva/mouth‐rinse cohorts independent of diagnosed periodontitis and in subgingival samples from patients with periodontitis.^47^, ^48^, ^49^, ^50^, ^51^, ^52^, ^53^ This dysbiosis may contribute to neuroinflammation, Aβ accumulation, and cognitive impairment through direct microbial invasion and systemic inflammatory pathways.^9^ While evidence supports the association between oral dysbiosis and AD, further research is needed to elucidate whether microbial alterations are causative or secondary to disease progression. Therapeutic strategies targeting oral microbial modulation, such as improved oral hygiene or probiotic interventions, may hold promise in mitigating AD risk (Figure 1).

## 4 SYSTEMIC DISEASES, ORAL MICROBIOTA, AND AD

Chronic systemic diseases, including type 2 diabetes, hypertension, chronic kidney disease (CKD), and autoimmune diseases, significantly exacerbate AD progression through overlapping pathological mechanisms.^54^ These conditions contribute to neurodegeneration by promoting chronic inflammation, oxidative stress, vascular dysfunction, and metabolic disturbances, all of which intersect with key AD pathways.

Emerging evidence suggests that oral microbiota may act as a modifiable risk factor, bridging systemic diseases and AD through microbial translocation, immune activation, and shared inflammatory pathways.^3^ Targeting oral dysbiosis (e.g., via improved dental hygiene or antimicrobial therapies) could represent a novel preventive strategy against AD in high‐risk populations.^55^

### 4.1 Type 2 diabetes oral microbiota and AD

Epidemiological studies indicate that type 1 diabetes mellitus (T2DM) significantly increases the risk of AD, with shared pathological mechanisms including insulin resistance (IR), oxidative stress, mitochondrial dysfunction, and impaired glucose metabolism in the brain.^56^, ^57^ T2DM and AD exhibit overlapping disruptions in insulin signaling pathways, particularly the phosphatidylinositol 3‐kinase (PI3K) and mitogen‐activated protein kinase (MAPK) cascades, which contribute to tau hyperphosphorylation and Aβ accumulation.^58^ Brain insulin resistance, termed “type 3 diabetes,” reduces glucose uptake via glucose transporters (GLUT‐1 and GLUT‐3), exacerbating neuronal hypometabolism.^59^ Mitochondrial dysfunction and oxidative stress further link the diseases, as reactive oxygen species (ROS) and advanced glycation end products (AGEs) promote neurodegeneration.^60^ Importantly, these same metabolic disturbances in T2DM also create an environment that disrupts the oral microbiota, potentially establishing a novel pathway connecting diabetes to neurodegeneration.

Diabetes mellitus (DM) significantly alters the composition of oral microbiota, often increasing pathogenic species while reducing beneficial ones. Studies show that DM promotes the growth of acidogenic and aciduric bacteria, particularly from the phylum *Firmicutes*, including *Streptococcus spp*., *Staphylococcus spp*., *Prevotella spp*., *Leptotrichia spp*., and *Veillonella spp*.^61^, ^62^ These bacteria thrive in hyperglycemic conditions, contributing to dental caries and periodontal disease.^63^ Additionally, *Candida albicans*, a fungal pathobiont, becomes more prevalent in diabetic patients due to elevated salivary glucose and immunosuppressive effects of DM.^64^ Conversely, some beneficial bacteria, such as *Proteobacteria* and *Bifidobacteria spp*., are reduced in diabetic individuals,^65^ disrupting microbial balance and increasing susceptibility to oral infections. Dysbiosis induced by DM converts commensal microbiota into pathogens, exacerbating oral diseases like periodontitis and candidiasis.^62^

Additionally, poorly controlled diabetes leads to a greater abundance of harmful bacteria, such as *P*. *gingivalis* and *T. denticola*, which are strongly associated with periodontal inflammation.^62^, ^66^ Reduced levels of protective species like *Proteobacteria* further impair oral immunity,^65^ facilitating pathogenic overgrowth. These changes highlight the bidirectional relationship between DM and oral dysbiosis, in which hyperglycemia drives microbial imbalance, and pathogenic bacteria worsen diabetic complications.^67^ Understanding these shifts may help develop targeted therapies to restore oral microbiota balance in diabetic patients.^62^

Metagenomic analysis of saliva and supragingival plaque from T2DM patients revealed enrichment of periodontal pathobionts, including *P*. *gingivalis*, *T. denticola*, and *F. nucleatum*. In contrast, there were no significant changes in the level of cariogenic species such as *S. mutans*.^68^ This selective shift in the microbial composition was accompanied by distinct metabolic changes, characterized by elevated levels of proinflammatory metabolites, including cadaverine and N, N‐dimethylarginine in a patient with T2DM. Notably, *P. gingivalis* abundance associated positively with cadaverine levels, indicating a non‐causal association consistent with community‐level dysbiosis, not direct production by *P. gingivalis*.^69^ The study also identified significant changes in ABC transporter pathways, which may contribute to the enhanced virulence of periodontal pathogens in diabetic conditions. These findings reveal that T2DM creates an oral microenvironment conducive to periodontal pathogenesis through both microbial population shifts and associated metabolic disturbances, providing potential biomarkers for early detection of oral complications in diabetic patients.^68^

Clinical studies revealed elevated levels of *F. nucleatum* (*Fn*) and *T. forsythia* (*Tf*) in both saliva and subgingival samples of diabetic patients compared to healthy controls, with these bacterial abundances positively correlating with fasting blood glucose and hemoglobin A1c (HbA1c) levels.^70^ Mechanistic studies showed that *Fn* culture filtrate (FNCF) activates the TLR2 signaling pathway in gingival epithelial cells, increasing pro‐inflammatory cytokines such as IL‐1β, IL‐6, and IL‐15, which are implicated in systemic insulin resistance.^70^ Furthermore, FNCF‐treated cell supernatants induced hepatic insulin resistance by upregulating extracellular signal‐regulated kinase (ERK) phosphorylation, which subsequently disrupted the insulin receptor substrate 1 (IRS1)/protein kinase B (AKT)/glycogen synthase kinase 3β (GSK3β) pathway, impairing glycogen synthesis in hepatocytes.^70^ Inhibition of ERK with U0126 reversed these effects, suggesting that ERK plays a critical role in mediating oral microbiota–induced metabolic dysfunction.^70^ These findings highlight how periodontal pathogens may exacerbate T2DM by promoting systemic inflammation and impairing hepatic glucose metabolism.

The study^71^ examined the salivary microbiota in patients with T2DM at different stages of periodontitis, identifying several important periodontal pathogens. In the saliva‐based dataset analyzed, the relative abundance of *Porphyromonas* was lower in stage IV periodontitis than in earlier stages. In mild to moderate disease (stages I–III), *P. gingivalis* is often readily detectable in saliva due to active bacterial shedding from inflamed pockets and gingival crevicular fluid. However, in advanced disease, despite a greater subgingival bacterial burden, salivary levels may appear lower. This counterintuitive finding reflects sampling limitations rather than a true reduction in *P. gingivalis* within the periodontal niche. As pocket depth increases, the subgingival environment becomes more anaerobic and anatomically isolated from the oral cavity, with *P. gingivalis* colonies migrating toward the pocket base, below the influence of salivary flow. In addition, epithelial thickening, ulceration, and reduced fluid exchange further limit bacterial release into saliva. Consequently, saliva primarily represents supragingival and shallow‐pocket communities and under‐represents deep‐pocket species such as *P. gingivalis*. Validation through subgingival plaque analysis, the gold‐standard matrix for assessing pocket microbiota, is therefore warranted.^71^ While *T. denticola* was not explicitly identified, the detection of *Spirochaetaceae* at the family level implies the possible presence of related species. *F. nucleatum* was not specifically reported, though *Fusobacteriota* were detected at the phylum level. The study did not mention *A. actinomycetemcomitans*, despite identifying Actinobacteria, which encompasses this genus. *Streptococcus* was highly abundant, though the analysis was unable to distinguish *S. salivarius* from other streptococcal species. *Neisseria spp*. was one of the dominant genera, while *Veillonella* was also prominently present, although species‐level confirmation of *V. parvula* was lacking. Notably, *Gemella spp*. showed a positive correlation with markers of periodontal inflammation, underscoring their potential role in disease progression.^71^ These findings highlight the complexity of the salivary microbiota in T2DM‐associated periodontitis. However, more reliable confirmation of the presence and roles of specific oral species would be obtained through full‐length 16S rRNA gene sequencing (e.g., PacBio HiFi) coupled with taxonomic assignment against eHOMD/HOMD.

The converging evidence from epidemiological, microbiological, and mechanistic studies paints a compelling picture of the interconnectedness among T2DM, oral dysbiosis, and AD. The shared pathophysiological pathways, including insulin resistance, chronic inflammation, and oxidative stress, create a vicious cycle in which T2DM‐driven oral dysbiosis exacerbates systemic inflammation, which in turn may accelerate neurodegenerative processes in AD.^72^ Key periodontal pathogens like *P*. *gingivalis* and *F. nucleatum* not only perpetuate oral disease but also contribute to systemic insulin resistance and neuroinflammation, potentially acting as bridging factors between these conditions.^73^ The identification of microbial signatures (e.g., elevated *Veillonella*) common to both diseases highlights the potential for oral microbiome profiling as a non‐invasive biomarker for early risk stratification.^74^ Critically, these findings highlight the importance of oral health management in T2DM patients as a potential strategy to mitigate AD risk. Future research should prioritize longitudinal studies to establish causal relationships and explore targeted interventions, such as precision probiotics or anti‐inflammatory therapies, that could simultaneously address oral dysbiosis, metabolic dysfunction, and neurodegenerative processes. By elucidating the oral–systemic–neuro axis, this emerging paradigm offers novel opportunities for interdisciplinary approaches to prevent and manage these increasingly prevalent chronic diseases.

**FIGURE 2:**
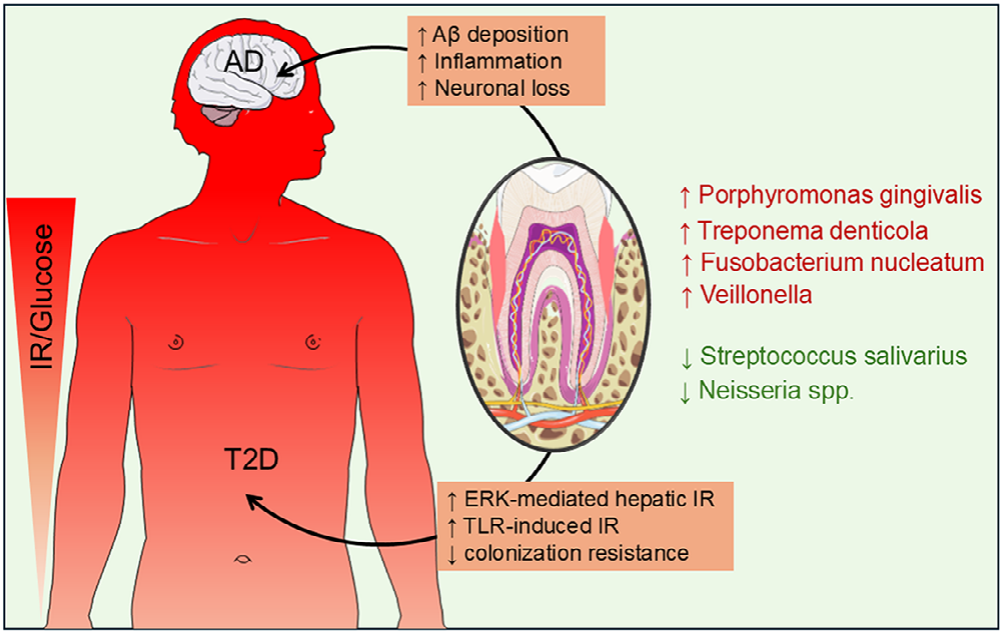
Interconnections among type 2 diabetes mellitus (T2DM), oral microbiome dysbiosis, and Alzheimer's disease (AD) pathogenesis. The diagram illustrates the tripartite relationship among T2DM, oral microbial shifts, and AD progression. T2DM induces oral dysbiosis characterized by increased periodontal pathogens (*Porphyromonas gingivalis, Treponema denticola, Fusobacterium nucleatum*, and *Veillonella)* and decreased commensals (*Streptococcus salivarius, Neisseria spp*.), promoting inflammation, amyloid beta (Aβ) deposition and neuronal loss. On the other hand, these pathogens increase extracellular signal‐regulated kinase (ERK) and Toll‐like receptor (TLR)–induced insulin resistance (IR) and decrease colonization resistance, creating a vicious cycle that links diabetic dysbiosis to AD progression.

In brief, T2DM‐associated oral dysbiosis overlaps with AD‐linked signatures (enrichment of periodontopathogenic consortia and loss of health‐associated commensals), aligning with systemic insulin resistance and neuroinflammatory pathways. These convergences nominate the oral cavity as a modifiable interface between metabolism and cognition. Prospective, species‐resolved, and interventional studies in T2DM are warranted to test whether improving oral ecology favorably shifts cognitive trajectories (Figure 2).

### 4.2 Hypertension, oral microbiota, and AD

Emerging evidence highlights hypertension as a significant risk factor for AD pathology, with mechanistic links involving both cerebrovascular dysfunction and neurodegenerative processes. A 2019 study in *Hypertension* demonstrated that individuals aged ≥ 55 years with hypertension exhibited higher levels of AD biomarkers, including Aβ plaques and neurofibrillary tau tangles. These pathological aggregates disrupt neuronal communication by impairing axonal transport and synaptic function, a process likened to the “trigger and bullet” of AD pathogenesis. Midlife hypertension (ages 40–64) has also been associated with increased dementia risk, suggesting a critical window for vascular contributions to neurodegeneration.^75^

The pathophysiological mechanisms linking hypertension to AD are multifactorial.^76^ Chronic hypertension promotes cerebral small vessel disease, which impairs perfusion and microvascular integrity.^76^ This vascular dysfunction may lead to hypoperfusion, BBB disruption, and subsequent failure of Aβ clearance, exacerbating protein accumulation.^77^ The compromised BBB further restricts glucose delivery while permitting neurotoxic infiltration, creating a feed‐forward cycle of neuroinflammation and vascular injury.^78^ Notably, hypertension‐associated strokes contribute to vascular dementia, though even subclinical vascular damage may accelerate AD pathology through ischemic and inflammatory pathways.^79^ Collectively, these findings highlight hypertension as a modifiable risk factor with both direct and indirect roles in AD progression.

Importantly, emerging research suggests these hypertension‐mediated pathways may be further exacerbated by oral microbiota dysbiosis, creating an additional biological link between hypertension and neurodegenerative pathology.^80^ A systematic review by Al‐Maweri et al.^81^ analyzed 17 studies comprising 6007 subjects to evaluate associations between oral microbiota and hypertension. While significant heterogeneity existed across studies in methodology and sample types, most studies (16/17) reported distinct oral microbial profiles in hypertensive versus normotensive individuals. Several bacterial taxa showed consistent enrichment in hypertensive patients across multiple studies, particularly when considering only those that controlled for false discovery rates and confounders. The genera *Atopobium, Prevotella*, and *Veillonella* were consistently elevated in hypertension across saliva samples from different populations.^81^

Periodontal pathogens such as *P*. *gingivalis*, *T. forsythia*, and *T. denticola* were frequently higher in subgingival plaque from individuals with hypertension.^82^, ^83^, ^84^ Other bacteria repeatedly linked to hypertension included *Aggregatibacter, Kingella, Lautropia*, and *Leptotrichia* in both saliva and subgingival samples.^82^ Interestingly, some nitrate‐reducing bacteria like *Neisseria* and *Rothia* showed conflicting results, being enriched in some studies but depleted in others.^82^, ^85^, ^86^

Recent research has shed light on the potential influence of oral microbiota on cardiovascular health, particularly in relation to blood pressure regulation. A study by LaMonte et al.^87^ examined the associations between subgingival plaque bacteria and hypertension risk in postmenopausal women, identifying several key microbial species that may play a role in blood pressure modulation. Among these, *P*. *gingivalis, T. denticola, F. nucleatum, S. salivarius, Neisseria spp., V. parvula*, and *Gemella spp*. emerged as particularly noteworthy due to their established links to periodontal disease and systemic inflammation.^88^ *P*. *gingivalis*, a well‐known periodontal pathogen, has been implicated in systemic inflammation and endothelial dysfunction, both of which are key contributors to hypertension development.^89^ While this study did not find a significant direct association between *P. gingivalis* and blood pressure categories, previous research has detected this bacterium in atherosclerotic plaques, suggesting it may contribute to vascular dysfunction through indirect mechanisms.^83^ Similarly, *T. denticola*, another major periodontal pathogen, was not significantly associated with blood pressure in this cohort, but its close relative *Treponema socranskii* showed enrichment in hypertensive women, indicating species‐specific associations within *Treponema* and the need for strain‐resolved, mechanistic studies before inferring shared cardiovascular pathways.^90^

Interestingly, different subspecies of *F. nucleatum* exhibited opposing relationships with blood pressure status. *F. nucleatum subsp. nucleatum* was more abundant in hypertensive women, while *F. nucleatum subsp. polymorphum* was enriched in normotensive individuals, suggesting strain‐specific effects on cardiovascular health.^91^, ^92^

Among the commensal bacteria examined, *S. salivarius* presented an unexpected finding. Typically considered a benign oral inhabitant, it was associated with an increased risk of hypertension in this study.^93^ This surprising result suggests that under certain conditions, typically harmless oral bacteria may contribute to blood pressure dysregulation, possibly through disruption of nitrate metabolism or promotion of low‐grade inflammation.^93^ In contrast, *Neisseria* species, particularly *Neisseria subflava*, emerged as potentially protective due to their nitrate‐reducing capabilities.^94^ These bacteria facilitate the conversion of dietary nitrate to nitrite, a precursor for nitric oxide (NO) production, which plays a crucial role in vasodilation and blood pressure regulation.^94^ The study found that a higher abundance of *N. subflava* was associated with reduced hypertension risk, supporting the importance of oral nitrate‐reducing bacteria in cardiovascular health.^95^

The study also examined *V. parvula*, a bacterium known for its ability to metabolize lactate into anti‐inflammatory propionate. While *V. parvula* itself was not directly analyzed, related *Veillonellaceae* species showed enrichment in hypertensive women, suggesting a possible, though complex, role in blood pressure modulation. Conversely, *Gemella morbillorum* was associated with lower hypertension risk, potentially through competitive exclusion of pathogenic bacteria or modulation of nitrate metabolism in the oral cavity.^87^ Although *G. morbillorum* lacks canonical nitrate reductase genes, it may indirectly influence oral nitrate metabolism by stabilizing biofilm microenvironments and supporting the metabolic activity of co‐resident nitrate‐reducing taxa such as *Neisseria* and *Veillonella*. Through these ecological interactions, *Gemella* contributes to maintaining the oral nitrate–nitrite–NO axis and vascular homeostasis.

These findings collectively highlight the complex interplay between oral microbiota and blood pressure regulation. While certain pathogenic bacteria appear to promote hypertension through inflammatory pathways, other commensal species may offer protection via nitrate metabolism and maintenance of microbial balance. The study by Lamonte et al.^87^ provides valuable epidemiological evidence supporting the oral microbiota's role in cardiovascular health, though mechanistic studies are needed to establish causal relationships. Future research should focus on elucidating the specific pathways through which these bacteria influence blood pressure, potentially opening new avenues for microbiota‐targeted interventions in hypertension prevention and management.

In a cohort study conducted in Qatar involving 1190 participants, the salivary microbiome profiles were found to differ based on blood pressure status. *T. socranskii* was more prevalent among individuals with hypertension, while distinct strain‐level patterns in *F. nucleatum* helped differentiate between groups: the subspecies *nucleatum* was higher in hypertensive participants, whereas the subspecies *polymorphum* was elevated in those with normal blood pressure.^86^ Additionally, levels of *Veillonellaceae* were also increased in individuals with hypertension, while nitrate‐reducing *Neisseria* species, such as *N. subflava*, were found to be more abundant in normotensive individuals.^96^, ^97^ Furthermore, *S. salivarius* was associated with a higher risk of developing hypertension, while *G. morbillorum* was linked to a lower risk.^87^, ^98^

These species‐ and strain‐specific signals motivate validation and careful translational study in hypertension management.^99^, ^100^ Further studies are needed to validate these associations and explore potential clinical applications of these discoveries in hypertension management strategies.

**FIGURE 3:**
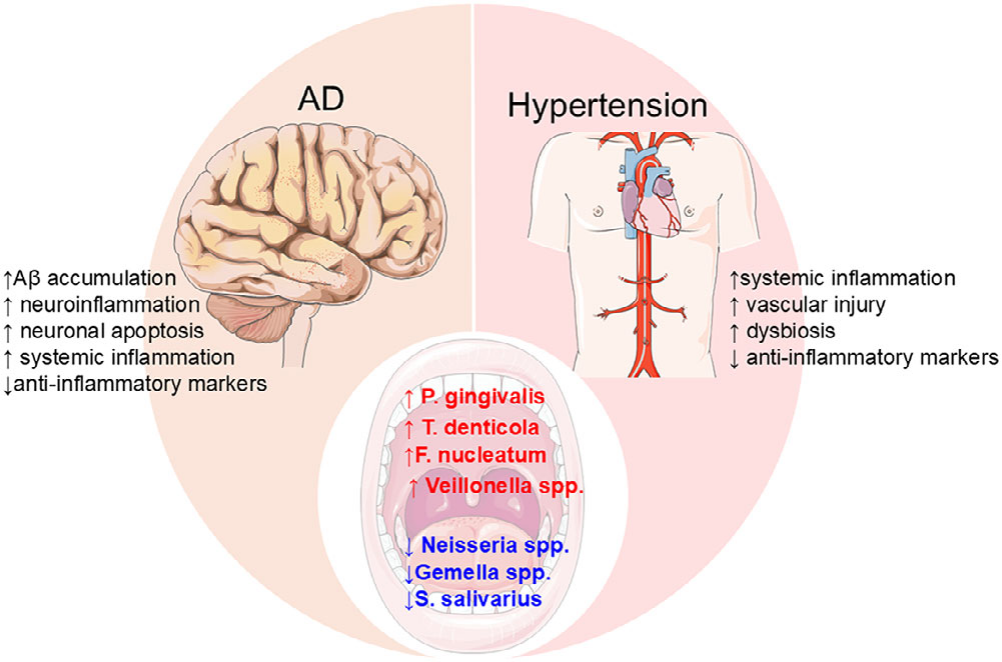
Hypertension, oral microbiome dysbiosis, and Alzheimer's disease (AD) interconnections. The diagram illustrates the tri‐directional relationship among hypertension, oral microbial shifts, and AD pathogenesis. Hypertension induces oral dysbiosis characterized by increased periodontal pathogens (*Porphyromonas gingivalis, Treponema denticola, Fusobacterium nucleatum*, and *Veillonella*) and decreased commensals (*Gemella spp, Streptococcus salivarius, Neisseria spp*.), promoting inflammation, amyloid beta (Aβ) deposition, and neuronal loss. On the other hand, these pathogens increase blood–brain barrier disruption and vascular injury, and reduce nitrate metabolism, creating a vicious cycle that links hypertension dysbiosis to AD progression

Emerging research reveals a striking overlap in oral microbiota alterations between AD and hypertension, suggesting shared microbial pathways that may contribute to both conditions.^101^ Key pathogenic bacteria, such as *P*. *gingivalis, T. denticola*, and *F. nucleatum*, are consistently elevated in both AD and hypertensive patients, implicating their role in promoting systemic inflammation, endothelial dysfunction, and neurodegeneration.^89^ These bacteria contribute to AD pathogenesis through Aβ accumulation and neuroinflammation, while in hypertension, they exacerbate vascular dysfunction via pro‐inflammatory cytokine release and impaired NO signaling.^102^ Conversely, protective commensals like *Neisseria spp. and Gemella spp*. are depleted in both conditions, potentially diminishing their anti‐inflammatory and nitrate‐reducing benefits. Notably, *S. salivarius* and *V. parvula* exhibit divergent associations; *S. salivarius* is reduced in AD but linked to hypertension risk, while *V. parvula* is elevated in both diseases, highlighting context‐dependent roles.^103^ The convergence of oral dysbiosis in AD and hypertension underscores the microbiota's dual impact on vascular and neurological health, suggesting that microbiota‐targeted interventions could simultaneously mitigate both conditions (Figure 3).

### 4.3 CKDs, oral microbiota, and AD

A growing body of evidence suggests a significant association between CKD and AD, as highlighted in the review by Zhang et al.^104^ Clinical observations indicate that CKD patients frequently experience cognitive decline and are more susceptible to developing AD, with some studies showing cognitive improvement after kidney transplantation.^105^ The authors propose several potential mechanisms linking these conditions, including uremic toxin accumulation, vascular dysfunction, and metabolic disturbances characteristic of CKD that may contribute to AD pathogenesis.^105^ Key pathological connections involve the role of indoxyl sulfate and other uremic toxins in promoting oxidative stress and inflammation, as well as disturbances in calcium–phosphate metabolism that may accelerate neurodegenerative processes.^104^ CKD comorbidities (anemia, blood‐pressure abnormalities, and vitamin D deficiency) each associate with cognitive impairment via plausible mechanisms (reduced cerebral oxygen delivery, cerebrovascular injury, neuroinflammation). In older adults with CKD, anemia has been linked to accelerated cognitive decline in prospective cohort analyses from CRIC.^106^ Likewise, in CKD populations, higher blood pressure variability is associated with greater risk of cognitive impairment/dementia, underscoring the vascular contribution to brain health.^107^ Observational data also connect low 25(OH)D with poorer cognitive performance in CKD (with effect modification by albuminuria and age/sex in some analyses), though interventional certainty remains limited.^108^, ^109^ Collectively, these primary studies support addressing CKD complications when evaluating cognition in CKD, while recognizing that causality remains unresolved and dedicated longitudinal/interventional work is needed.^104^ This comprehensive analysis highlights the importance of considering kidney–brain axis interactions in both the prevention and management of cognitive decline in CKD patients. Intriguingly, CKD also induces profound shifts in the oral microbiota, creating a potential bidirectional link between kidney dysfunction, microbial dysbiosis, and neurodegeneration.

The oral microbiota undergoes significant compositional changes in patients with CKD.^110^ CKD leads to the accumulation of uremic toxins, including urea, which alters the oral microbial environment by increasing pH and favoring certain bacterial taxa.^111^ In vitro studies using constant‐depth film fermenters (CDFFs) have demonstrated that elevated urea concentrations, mimicking CKD conditions, influence microbial diversity and abundance, with shifts observed in genera such *as Fusobacterium, Neisseria, Streptococcus*, V*eillonella*, and *Porphyromonas*.^110^ The study showed that *Porphyromonas, Fusobacterium*, and *Veillonella* increased in mimicked CKD conditions; however, *Streptococcus* and *Neisseria* decreased.^110^ The study highlights the complex interplay among urea levels, pH, and microbial succession, emphasizing the need for further research on long‐term microbiota recovery post‐transplantation and its clinical implications for CKD and transplant patients.^110^

The study by Yasuno et al.^112^ investigated oral microbiota alterations in CKD patients across different disease stages using 16S rRNA gene sequencing. The analysis revealed significant shifts in microbial composition associated with CKD progression. At the genus level, patients with CKD stages three to five exhibited increased abundance of periodontal pathogens, including *Tannerella, Fusobacterium*, and *Capnocytophaga*, compared to earlier CKD stages. The study also identified qualitative and quantitative changes in bacterial communities through β‐diversity assessments, with more pronounced differences observed in later CKD stages. The increased prevalence of periodontal pathobionts in CKD patients aligns with previous reports of higher periodontal disease incidence in this population.^113^, ^114^ The findings particularly highlight the enrichment of periodontal pathogens *Tannerella* and *Fusobacterium* in advanced CKD stages, which may contribute to the oral–systemic inflammatory burden in these patients.^115^, ^116^

The study by Hu et al.^117^ provides compelling evidence for an association between the oral microbiota and CKD. Analyzing samples from multiple oral sites in 77 participants, the researchers identified significant differences in microbial composition between CKD and non‐CKD individuals, particularly in saliva and anterior mandibular lingual samples. Notably, CKD patients exhibited enrichment of *Neisseria* (Proteobacteria phylum) and depletion of *Veillonella* and *Streptococcus* (Firmicutes phylum). These patterns persisted even after adjusting for comorbidities such as diabetes and hypertension. The microbial alterations correlated with clinical markers of kidney function, with higher *Neisseria* and lower *Streptococcus* abundances associating with reduced estimated glomerular filtration rate (eGFR). Importantly, *Neisseria* abundance also showed a positive correlation with plasma IL‐18 levels, suggesting a potential link between oral dysbiosis and systemic inflammation in CKD pathogenesis.^117^

The study further demonstrated the diagnostic potential of oral microbiome profiling for CKD. Specific bacterial ratios, particularly the *Neisseria*‐to‐*Veillonella* ratio in anterior lingual samples and the *Neisseria*‐to‐*Streptococcus* ratio in saliva, showed promising accuracy in distinguishing CKD patients from controls.^117^ Predictive metagenomic analysis revealed functional alterations in CKD‐associated oral microbiota, including disruptions in lipid metabolism and inflammatory pathways, which may contribute to disease progression. However, the study's cross‐sectional design and relatively small sample size (*n* = 18 CKD patients) limit causal inferences and generalizability.^118^ Additionally, potential confounding factors such as dietary habits and medication use were not fully accounted for in the analysis. Despite these limitations, the findings suggest that oral microbiota characterization could serve as a non‐invasive approach for CKD risk assessment and highlight the need for longitudinal studies to examine whether oral microbial modulation might influence CKD outcomes.

The implications of these findings extend beyond diagnostics, raising important questions about potential mechanisms linking oral bacteria to kidney health. The observed associations between specific oral taxa and inflammatory biomarkers suggest that oral–gut–kidney axis interactions may play a role in CKD progression.^119^ Future research should investigate whether targeted oral microbiota interventions could modify CKD risk or progression and explore how systemic conditions like CKD might reciprocally shape the oral ecosystem.^120^ Larger, prospective studies incorporating detailed clinical metadata and multi‐omics approaches will be essential to validate these findings and elucidate the complex interplay among oral microbiota, systemic inflammation, and kidney function.^121^

The study by Randall et al.^122^ demonstrates that CKD induces significant taxonomic shifts in the oral microbiota that contribute to periodontal pathology. In rodent models of CKD, researchers observed a consistent depletion of health‐associated commensal bacteria, particularly *Streptococcus* and *Rothia* species.^122^ These changes occurred alongside a proportional expansion of Gram‐negative *Proteobacteria*.^122^ The dysbiotic state also featured the emergence of typically rare taxa such as *Acinetobacter*, which was completely absent in control animals but increased in CKD subjects.^122^

The altered microbial composition appears driven by CKD‐induced changes in the oral environment, particularly elevated salivary urea levels.^110^ Urease‐producing species demonstrated a clear growth advantage under these conditions, while acid‐producing commensals like streptococci were disadvantaged.^123^ Longitudinal analysis revealed the dysbiosis progressed over time, with increasing microbial heterogeneity and the appearance of potentially pathogenic species such as *Psychrobacter*.^124^ Importantly, the study showed this dysbiotic community could be transmitted to germ‐free recipients, where it reproduced the periodontal bone loss phenotype despite normal kidney function in the new hosts.^122^ The findings suggest that CKD creates an oral microenvironment that selects for urea‐tolerant, often pathogenic species while depleting protective commensals, ultimately disrupting periodontal bone homeostasis through mechanisms distinct from classical inflammatory periodontitis.^125^ This microbial shift may represent a key mediator in the development of CKD‐associated periodontal disease.

**FIGURE 4:**
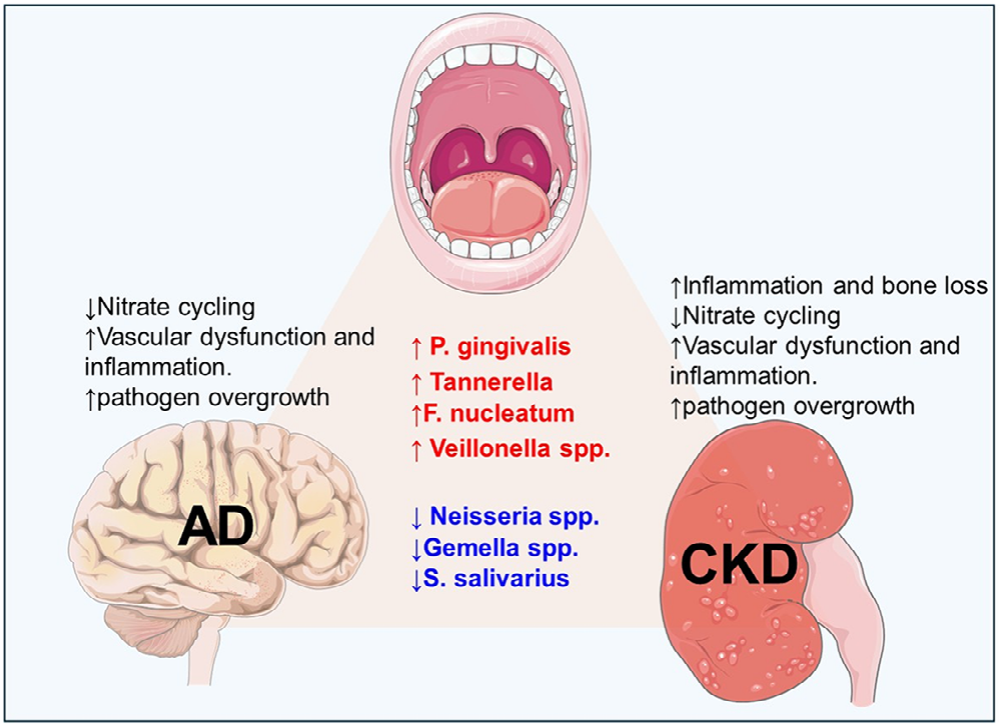
Chronic kidney diseases (CKDs), oral microbiome dysbiosis, and Alzheimer's disease (AD) interconnections. The diagram illustrates the tri‐directional relationship among CKD, oral microbial shifts, and AD pathogenesis. CKD induces oral dysbiosis characterized by increased periodontal pathogens (*Porphyromonas gingivalis, Tannerella, Fusobacterium nucleatum*, and *Veillonella*) and decreased commensals (*Gemella spp, Streptococcus salivarius, Neisseria spp*.), promoting inflammation, nitric cycling damage, and vascular dysfunction, creating a vicious cycle that links CKD dysbiosis to AD progression

Emerging research reveals a striking overlap in oral microbiota alterations between AD and CKD, suggesting shared microbial pathways that may contribute to both conditions.^112^ Key periodontal pathogens, *P. gingivalis*, *F. nucleatum*, and *Veillonella*, are consistently elevated in both diseases, promoting systemic inflammation, Aβ accumulation (in AD), and uremic toxin‐driven dysbiosis (in CKD).^126^ These bacteria contribute to neurodegeneration via gingipains, LPS‐induced inflammation, and BBB disruption in AD. In CKD, they thrive in the urea‐rich oral environment, exacerbating periodontal disease and systemic inflammation.^126^ Conversely, protective commensals like *S. salivarius* and *Neisseria spp*. are depleted in both AD and CKD, impairing microbial homeostasis, nitrate metabolism, and anti‐inflammatory defenses.^126^ Notably, *Neisseria*, which supports NO production, is reduced in both conditions, potentially worsening vascular dysfunction, a key link between CKD and AD.^127^ Additionally, *Gemella spp*., associated with cognitive protection in AD, may also decline in CKD, further amplifying systemic inflammation.^128^ This shared dysbiotic profile suggests that oral microbiota disruption may serve as a critical intersection point between kidney dysfunction and neurodegeneration, highlighting potential therapeutic strategies targeting oral pathogens/pathobionts to mitigate both diseases (Figure 4).

### 4.4 Autoimmune diseases, oral microbiota, and AD

A large‐scale analysis of electronic health records from > 300,000 patients at University of California San Francisco and Stanford revealed a significant association between autoimmune disorders and an increased risk of AD.^129^ The study found that individuals with autoimmune conditions had 40% to 70% higher AD risk, with consistent risk elevations across multiple study designs (odds ratios ranging from 1.4 to 1.7).

These findings strengthen the connection between immune dysregulation and AD pathogenesis, implicating chronic inflammation or other immune‐mediated mechanisms. The study highlights the potential for autoimmune disorders to serve as risk markers for AD and highlights the need for further research into shared biological pathways. This could eventually inform early detection strategies and targeted interventions for at‐risk populations.

Growing evidence implicates oral dysbiosis in the pathogenesis of autoimmune disorders.^130^, ^131^, ^132^ Experimental periodontitis models demonstrate that microbial shifts can trigger localized Th17 cell activation,^133^ a key driver of autoimmunity.

Notably, *P. gingivalis* contributes to rheumatoid arthritis pathogenesis through its peptidylarginine deiminases (PADs), which generate citrullinated autoantigens,^134^ stimulating anti‐citrullinated peptide antibody production and complement activation.^135^ Similarly, *A. actinomycetemcomitans* exacerbates rheumatoid arthritis (RA) via leukotoxin‐A‐mediated neutrophil hypercitrullination.^136^ In systemic lupus erythematosus, elevated serum antibodies against oral pathogens, including *A. actinomycetemcomitans* and *P. gingivalis*, correlate with disease activity,^137^ highlighting the oral microbiota's systemic immunomodulatory effects.

Emerging evidence suggests that specific alterations in the oral microbiota are associated with various autoimmune diseases, including spondyloarthritis, multiple sclerosis (MS), and primary biliary cholangitis (PBC)^138^, ^139^ demonstrating that patients with spondyloarthritis exhibit significant increases in salivary *Rothia mucilaginosa* and plaque‐derived *Fusobacterium* species, suggesting that spondyloarthritis may be influenced by oral microbial dysbiosis. These findings position the oral microbiota as a potential contributor to the pathogenesis of spondyloarthritis through mechanisms that remain to be fully elucidated.

Similarly, distinct microbial signatures have been identified in MS.^140^ Studies have shown that the salivary microbiota of MS patients is characterized by elevated abundances of *Staphylococcus, Actinomyces, Fusobacterium, Bacteroides, P. gingivalis*, *Prevotella, Veillonella*, and *Propionibacterium*, along with various uncultivable bacterial species.^141^ Of particular interest is Lipid 654, a metabolite produced by *P. gingivalis* that acts as a TLR2 ligand.^142^ This microbial product may promote inflammatory responses, potentially exacerbating neuroinflammatory processes in MS. These observations suggest that oral microbiome analysis could serve as a valuable tool for understanding MS pathogenesis and developing targeted therapeutic strategies.

In primary biliary cholangitis, significant shifts in the oral microbiota have also been documented. Abe et al.^143^ reported increased levels of *Eubacterium* and *Veillonella*, along with decreased *Fusobacterium*, in saliva samples from PBC patients. Further characterization using 16S rDNA sequencing revealed enrichment of *Bacteroidetes, Campylobacter, Prevotella*, and *Veillonella*, while *Enterococcaceae, Granulicatella, Rothia*, and *Streptococcus* were depleted in these patients.^144^ These microbial alterations may contribute to the inflammatory milieu characteristic of PBC, although the precise mechanisms linking oral dysbiosis to liver autoimmunity require further investigation.

Collectively, these findings highlight the growing recognition of oral–gut–systemic axis interactions in autoimmune diseases. The consistent demonstration of disease‐specific oral microbiota signatures across different conditions suggests that microbial profiling may have diagnostic and prognostic value, while also revealing potential therapeutic targets for modulating autoimmune responses.

A case–control study by Liu et al. in 2020^145^ investigated the potential link between subgingival microbiota composition and both periodontitis (PD) and RA in Chinese populations. The study compared subgingival plaque samples from 54 RA patients, 45 PD patients, and 44 healthy controls using Illumina MiSeq sequencing technology. While alpha diversity analysis revealed similar microbiome profiles across all three groups, significant compositional differences emerged at specific taxonomic levels. The researchers found that *Treponema* species were significantly more abundant in both PD and RA groups compared to healthy controls across all taxonomic levels from phylum to genus. Additionally, the RA group showed an increased abundance of *Porphyromonas, Prevotella*, and *Veillonella*, while demonstrating decreased levels of *Streptococcus, Gemella*, and *Planobacterium* compared to other groups.

Of particular interest was the identification of *Spirochaetes* as a potential microbial link between PD and RA pathogenesis.^145^ Although the study found no significant differences in overall microbial diversity or richness between groups, the distinct patterns of bacterial abundance suggest that specific oral microorganisms may contribute to the shared pathological mechanisms of these two conditions. These findings provide valuable insights for future research exploring the role of oral microbiota in autoimmune disease development and progression.

The study's limitations include its case–control design and relatively small sample size, which preclude causal inferences.^145^ However, the results contribute to growing evidence that oral microbial dysregulation may represent an important environmental factor in RA pathogenesis, potentially mediated through shared immunological pathways with periodontal disease. Future longitudinal studies could help clarify whether these microbial changes precede disease onset or result from established pathology.

A 2021 study published in *mSystems* investigated the salivary microbiome profile in patients with ankylosing spondylitis (AS), revealing significant differences compared to healthy controls.^144^ The research team found that AS patients exhibited distinct microbial alterations, such as a depletion of beneficial *Streptococcus* and enrichment of potentially pathogenic taxa, including *Veillonella*, *Brucella*, and *Campylobacter* concisus.^146^ These microbial changes were accompanied by elevated levels of 16 proinflammatory cytokines in saliva, including IL‐6 receptor α, IL‐2, IL‐10, and various interferons, along with increased concentrations of harmful compounds like cadaverine and putrescine.^144^

A clinical study^141^ investigated potential differences in oral microbiota composition between MS patients and healthy controls. The research team collected saliva samples from 30 MS patients and 30 healthy individuals, using both culture‐based methods and molecular techniques, including denaturing gradient gel electrophoresis (DGGE) to analyze bacterial populations.

The results revealed significant differences in oral microbial communities between the two groups. MS patients showed increased abundance of several bacterial genera including *Staphylococcus, Actinomyces, Fusobacterium, Bacteroides, Porphyromonas, Prevotella, Veillonella*, and *Propionibacterium*, along with various uncultivable bacterial strains.^147^ In contrast, healthy controls demonstrated higher prevalence of *Lactobacillus* and *Peptostreptococcus* species.^148^

Emerging research reveals a striking convergence in oral microbiota alterations between AD and autoimmune disorders, highlighting shared microbial signatures that may contribute to both conditions through chronic inflammation and immune dysregulation.^149^ Key periodontal pathogens, *P. gingivalis, T. denticola, F. nucleatum*, and *V. parvula*, are consistently elevated in both AD and autoimmune diseases (e.g., RA, MS, AS). These bacteria promote systemic inflammation via gingipains (*P. gingivalis*), LPS (*F. nucleatum*), and citrullinated autoantigens (*T. denticola*), which exacerbate neurodegeneration (Aβ accumulation in AD) and autoimmunity (Th17 activation in RA/MS). Conversely, protective commensals like *S. salivarius, Gemella spp*., and *Neisseria spp*. are depleted in both conditions, impairing microbial homeostasis and anti‐inflammatory defenses. Notably, *Streptococcus* species, which modulate immune tolerance, are reduced in AD, RA, and MS, while *Veillonella*, a genus linked to pro‐inflammatory cytokine production, is elevated across these diseases. This shared dysbiotic profile suggests that oral microbiota disruption may serve as a common environmental trigger for both neurodegeneration and autoimmunity, potentially through TLR2/4 activation, molecular mimicry, or compromised gut–brain–immune axis communication (Table 1).

**TABLE 1:** Systemic diseases, oral microbiome dysbiosis, and AD interconnections. The table illustrates the common bacterial trend, principal molecular/inflammatory mediators, and mechanistic links to AD across major systemic conditions.

Bacterial species	Trend in AD	Trend in autoimmune disorders	Potential shared mechanisms	References
*Porphyromonas gingivalis*	Increased	↑ (RA, MS; citrullination, TLR2 activation)	Gingipains promote Aβ & autoantigen production	Dominy et al.^30^; Curran et al.^134^
*Treponema denticola*	Increased	↑ (RA, PD; Th17 activation)	Triggers autoantibodies via hypercitrullination	Riviere et al.^32^; Konig et al.^136^
*Fusobacterium nucleatum*	Increased	↑ (MS, AS; pro‐inflammatory cytokines)	LPS disrupts immune tolerance & BBB integrity	Luo et al.^34^; Zangeneh et al.^141^
*Veillonella*	Increased	↑ (RA, MS; IL‐6/IL‐17 production)	Promotes Th17 polarization & neuroinflammation	Guo et al. (2021); Stoll et al.^22^
*Streptococcus salivarius*	Decreased	↓ (RA, MS; immune dysregulation)	Reduction impairs mucosal immunity & tolerance	Fu et al. (2022); PMID: 32565704
*Gemella spp*.	Decreased	↓ (RA; anti‐inflammatory role)	Depletion exacerbates inflammation	Adnan et al.^46^; PMID: 32565704
Neisseria spp.	Decreased	↓ (PBC; nitrate metabolism)	Loss reduces nitric oxide, worsening vascular/immune health	Liu et al.^37^; Abe et al. (2018)

Abbreviations: Aβ, amyloid beta; AD, Alzheimer's disease; AS, ankylosing spondylitis; IL, interleukin; MS, multiple sclerosis; LPS, lipopolysaccharide; PBC, primary biliary cholangitis; PD, periodontitis; RA, rheumatoid arthritis; TLR, Toll‐like receptor.

## 5 THERAPEUTIC POTENTIAL: TARGETING THE ORAL MICROBIOTA FOR AD PREVENTION AND TREATMENT

The growing recognition of the oral microbiota's role in AD pathogenesis has spurred interest in developing microbiota‐targeted interventions as potential preventive or therapeutic strategies.^150^ Current approaches to modulate oral microbial communities include probiotics and prebiotics, which aim to restore a healthy microbial balance.^151^ Probiotics such as *Lactobacillus* and *Bifidobacterium* have shown preliminary promise in reducing periodontal pathogens/pathobionts and suppressing inflammation in preclinical studies, while prebiotics like xylitol may selectively promote beneficial bacteria.^152^ However, translating these findings to AD‐specific applications requires further investigation, particularly regarding optimal strain selection, delivery methods, and long‐term efficacy.

Antimicrobial therapies represent another avenue for intervention, with both traditional antibiotics and novel antimicrobial peptides under exploration.^153^ While broad‐spectrum antibiotics can temporarily suppress pathogenic/pathobionts bacteria, their non‐specific nature risks disrupting commensal microbiota and promoting resistance.^154^

However, their long‐term use may exacerbate dysbiosis and increase resistance, underscoring the need for more targeted therapies. More targeted approaches, such as localized delivery of antimicrobial peptides or phage therapy against specific periodontal pathogens, may offer greater precision.^155^, ^156^ Preclinical studies show that inhibiting *P. gingivalis* gingipains can reduce bacterial burden, thereby reducing systemic inflammation and neuroinflammatory/amyloid readouts. An earlier gingipain inhibitor program in AD (atuzaginstat/COR388) was halted after a US Food and Drug Administration (FDA) clinical hold for liver safety; however, a Phase 2 trial of a next‐generation Kgp inhibitor (LHP588) in Pg‐positive AD is ongoing (NCT06847321; https://clinicaltrials.gov/study/NCT06847321?utm_source =). This approach is pathogen/virulence‐directed rather than a broad microbiota therapy, and clinical efficacy remains to be established.^30^

Lifestyle interventions remain a cornerstone of oral microbiota modulation, with improved oral hygiene practices and dietary modifications showing systemic benefits.^157^ Regular professional dental care, proper brushing techniques, and the use of antimicrobial mouthwashes can significantly reduce pathogenic bacterial loads. Dietary interventions rich in polyphenols, omega‐3 fatty acids, and fiber may also foster a healthier oral microbiota while concurrently reducing inflammation, a strategy that aligns with emerging research on the gut–brain axis.^158^

The development of novel therapies faces several challenges, including the complexity of host–microbiota interactions, individual variability in microbial communities, and the need for BBB penetration. Additionally, the bidirectional relationship between oral dysbiosis and systemic inflammation complicates targeted interventions, as modulating one aspect may have unpredictable effects on interconnected systems. Despite these hurdles, advancing technologies in microbiome sequencing, targeted drug delivery, and personalized medicine offer promising avenues to overcome current limitations and harness the oral microbiota's therapeutic potential for AD management.

Future research should prioritize longitudinal clinical trials to establish causality, optimize intervention timing, and evaluate combinatorial approaches that address both oral and systemic contributors to neurodegeneration. By integrating oral health into broader AD prevention strategies, this emerging field may open new frontiers in combating this devastating disease.

## 6 FUTURE DIRECTIONS AND CLINICAL IMPLICATIONS

The emerging connection between oral microbiota dysbiosis and AD opens promising avenues for research and clinical intervention, yet critical gaps remain in establishing causality and translating findings into effective therapies. A key priority is the initiation of large‐scale, longitudinal cohort studies to determine whether oral dysbiosis precedes and contributes to AD pathogenesis or merely reflects disease‐related changes in immunity and hygiene. Such studies should incorporate serial cognitive assessments, detailed oral health evaluations, and metagenomic sequencing to track temporal relationships between microbial shifts and neurodegeneration. Parallel animal models using fecal microbiota transplantation (FMT) from AD patients or germ‐free systems could help isolate the oral microbiota's mechanistic role.

The development of non‐invasive biomarkers represents another crucial direction. Salivary or subgingival plaque–based microbial signatures, combined with inflammatory markers like IL‐1β or gingipain activity, could stratify AD risk years before clinical symptoms emerge. Salivary or subgingival plaque‐based microbial and inflammatory profiles could potentially stratify individuals at elevated AD risk decades before symptom onset, particularly because subclinical periodontal dysbiosis often begins in early adulthood. Advances in multi‐omics technologies (metagenomics, metabolomics, proteomics) may enable the identification of predictive microbial consortia or pathogen‐derived metabolites (e.g., short‐chain fatty acids, LPS) linked to neuroinflammation. These biomarkers could be integrated with existing diagnostic tools, such as amyloid positron emission tomography or blood phosphorylated tau assays, to refine early detection paradigms.

Clinically, these insights advocate for the integration of oral health into AD prevention guidelines. Given the modifiability of oral dysbiosis through routine dental care, public health initiatives should emphasize periodontal treatment for high‐risk populations (e.g., apolipoprotein E ε4 carriers, diabetics). Collaborative care models that unite neurologists and dentists could optimize interventions, from professional plaque removal to antimicrobial therapies, while also monitoring cognitive outcomes. Notably, ongoing trials testing gingipain inhibitors may pave the way for FDA‐approved, microbiota‐targeted AD therapies if efficacy is demonstrated. It is noteworthy that although gingipain inhibitors such as COR388 (atuzaginstat) initially demonstrated therapeutic promise against *P. gingivalis*–associated neurodegeneration, their clinical development was discontinued in 2022 after reports of hepatotoxicity.

Personalized medicine approaches hold particular promise. Machine learning algorithms analyzing individual microbiome profiles, genetic risk factors (e.g., *TREM2* variants), and lifestyle data could tailor interventions, such as probiotic regimens or dietary adjustments, to mitigate patient‐specific dysbiosis.^159^ For instance, patients with dominant *P. gingivalis* loads might receive targeted antimicrobials, while those with depleted commensals could benefit from prebiotic supplements. However, challenges like microbiota variability, BBB penetration of therapeutics, and the need for standardized sampling protocols must be addressed to realize this precision medicine vision.

Ultimately, unraveling the oral microbiota–AD axis demands interdisciplinary collaboration across neurology, microbiology, and dentistry. By prioritizing causal research, biomarker innovation, and integrated care models, this field may yield transformative strategies to delay or prevent AD, a paradigm shift from treatment to early interception rooted in systemic health.

## 7 CONCLUSION

The accumulating body of evidence underscores a compelling association between oral microbiota dysbiosis and AD, mediated through interconnected pathways of chronic inflammation, microbial translocation, and systemic metabolic dysfunction. Key findings from clinical and preclinical studies reveal consistent alterations in the oral microbiota of AD patients, characterized by an enrichment of periodontal pathogens such as *P. gingivalis*, *T. denticola*, and *F. nucleatum*, alongside depletion of health‐associated commensals. While direct causality remains to be established, the consistent correlation among oral dysbiosis, neuroinflammatory biomarkers, and cognitive decline highlights the importance of oral health as a modifiable contributor to AD risk.^160^

Critically, systemic diseases, including diabetes, hypertension, periodontitis, and cardiovascular disease (CVD), exacerbate this relationship by amplifying inflammatory and vascular insults to the brain. For instance, diabetes‐induced insulin resistance and hypertension‐driven hypoperfusion synergize with oral dysbiosis to disrupt the BBB, facilitate microbial neuroinvasion, and accelerate neuronal damage. Periodontitis serves as a chronic reservoir of pro‐inflammatory mediators and pathogens that may directly or indirectly fuel neurodegenerative processes. This triad of oral–systemic–neural interactions highlights the need to view AD through a holistic lens, in which peripheral health significantly influences central nervous system integrity.

To translate these insights into clinical benefits, a concerted call to action is imperative, and interdisciplinary research and targeted clinical trials are urgently needed. First, interdisciplinary collaboration among neurologists, microbiologists, and dentists must be prioritized to unravel causal mechanisms and identify actionable therapeutic targets. Large‐scale longitudinal studies are urgently needed to validate whether oral microbiota modulation, via probiotics, antimicrobials, or gingipain inhibitors, can delay AD onset or progression. Second, clinical trials should explore combinatorial approaches targeting both oral and systemic health, such as integrating periodontal therapy with anti‐inflammatory diets in high‐risk populations. Finally, public health initiatives must emphasize oral hygiene as a modifiable dementia risk factor, particularly in aging populations with comorbid metabolic or vascular conditions.

In conclusion, the oral microbiota represents a promising yet underexplored frontier in AD research. By bridging gaps between oral health and neurodegeneration, this paradigm offers innovative opportunities for early intervention, personalized treatment, and ultimately, disease prevention. The path forward demands rigorous science, clinical innovation, and a reimagining of AD not just as a brain‐limited disorder, but as a systemic condition in which the mouth may hold unexpected keys to preserving cognitive resilience (Table S1 in supporting information).

## CONFLICT OF INTEREST STATEMENT

All authors declare that they have no competing interests. Author disclosures are available in the supporting information.

## Supporting information



Supporting Information

Supporting Information
