# Supplementary material for: Unraveling the oral microbiome's role in Alzheimer's disease: From pathophysiology to therapeutic potential
Source: Alzheimers Dement. 2025 Dec 13;21(12):e71011. doi: 10.1002/alz.71011 (PMC12701368; doi:10.1002/alz.71011)
Supplement: Supplementary file 2 — Supporting Information [file ALZ-21-e71011-s001.docx]

**Unraveling the Oral Microbiome's Role in Alzheimer's Disease: From Pathophysiology to Therapeutic Potential**

Gilliana Rozenblum^1^, Karima Ait-Aissa^1^, Gadeer Zahran^1^, Mahdieh Alipour^1^, Amal M. Sahyoun^1^, Undral Munkhsaikhan ^1^, Adam Kassan^2^, Tauheed Ishrat^3^, Qi Wang^1^, Ammaar Abidi^1*^, Modar Kassan^1*^

1. College of Dental Medicine, Lincoln Memorial University, LMU Tower, 1705 St. Mary Street, Knoxville, TN 37917, USA.
2. School of Pharmacy, West Coast University, 590 N. Vermont Ave, Los Angeles, CA, 90004, USA
3. Department of Anatomy and Neurobiology, University of Tennessee Health Science Center, Memphis, TN, USA; Department of Pharmaceutical Sciences, College of Pharmacy, The University of Tennessee Health Science Center, Memphis, TN, United States; Neuroscience Institute, University of Tennessee Health Science Center, Memphis, TN, USA

^*^Corresponding Authors: Ammaar Abidi ([Ammaar.abidi@lmunet.edu](mailto:Ammaar.abidi@lmunet.edu)) and Modar Kassan ([modar.kassan@lmunet.edu](mailto:modar.kassan@lmunet.edu)). LMU Tower, 1705 St. Mary Street, Knoxville, TN 37917, USA.

1. **Literature Search Strategy**

A comprehensive and systematic literature search was conducted across three major databases, PubMed, Scopus, and Web of Science, to identify relevant studies published between January 2010 and April 2025. The search strategy incorporated both Medical Subject Headings (MeSH) and free-text keywords related to the oral microbiome and neurodegenerative disorders, including “oral microbiome,” “Alzheimer’s disease,” “periodontal pathogens,” and “neuroinflammation.” Boolean operators (AND, OR) were used to refine the search and capture studies examining associations between oral microbial communities, neuroinflammatory processes, and Alzheimer’s disease pathology.

All retrieved records were imported into reference management software for organization and duplicate removal. Following deduplication, the remaining studies were screened systematically by title and abstract to determine their relevance based on predefined eligibility criteria.

**Inclusion and Exclusion Criteria**

Studies were included if they:

- Investigated the relationship between the oral microbiota and neuroinflammatory or neurodegenerative mechanisms.
- Reported primary data, systematic reviews, or methodological resources relevant to Alzheimer’s disease and periodontal interactions.
- Were published in English in peer-reviewed journals.

Studies were excluded if they:

- Were non-English publications.
- Represented conference abstracts without a full manuscript.
- Consisted of editorials, commentaries, or other non-peer-reviewed sources.
- Did not directly address oral–neural or microbiome–neuroinflammatory pathways.

**Screening and Selection Process**

Two independent reviewers conducted the screening process to assess study eligibility. Any discrepancies were resolved through discussion and consensus. After applying all inclusion and exclusion criteria, the studies meeting the final selection parameters were incorporated into the qualitative synthesis, which comprised a combination of original research, review articles, and methodological or repository papers describing analytical approaches and data resources relevant to oral–brain interaction research.

**Table Supplement 1. Systemic conditions associated with oral microbiota dysbiosis and their mechanistic links to Alzheimer’s disease (AD).**
This table summarizes the major systemic disorders that influence oral microbial composition and contribute to AD pathogenesis through inflammatory, metabolic, and vascular pathways. “↑” and “↓” indicate increased or decreased bacterial abundance, respectively. Each condition demonstrates distinct oral microbial shifts accompanied by key molecular mediators (cytokines, oxidative stress markers, metabolic regulators) that collectively promote neuroinflammation, endothelial dysfunction, and amyloid-β/tau pathology. Together, these data highlight the oral–systemic–neural axis as a convergent pathway in AD progression.

| **Systemic Condition** | **Key Oral Microbial Changes** | **Principal Molecular/Inflammatory Mediators** | **Mechanistic Link to Alzheimer’s Disease** |
| --- | --- | --- | --- |
| Type 2 Diabetes Mellitus (T2DM) | ↑ Porphyromonas gingivalis, Treponema denticola, Fusobacterium nucleatum, Veillonella spp.  ↓ Neisseria, Proteobacteria | ↑ IL-1β, IL-6, TNF-α, ROS, AGEs; Insulin resistance (PI3K/MAPK pathways), Mitochondrial dysfunction, Gingipains | Chronic hyperglycemia promotes oral dysbiosis → systemic inflammation → microglial activation and impaired insulin signaling in the brain (“type 3 diabetes”) → ↑ Aβ and tau phosphorylation |
| Hypertension | ↑Porphyromonas gingivalis, Treponema denticola, Fusobacterium nucleatum, Veillonella parvula  ↓ Nitrate-reducing Neisseria, Gemella morbillorum | ↑ TNF-α, IL-6, CRP; ↓ Nitric oxide (NO) bioavailability; Endothelial dysfunction; Oxidative stress (ROS) | Vascular injury and BBB disruption reduce cerebral perfusion → impaired Aβ clearance → neuroinflammation and neurovascular dysfunction; oral nitrate loss decreases NO-mediated vasoprotection |
| Chronic Kidney Disease (CKD) | ↑Tannerella, Fusobacterium, Porphyromonas, Veillonella  ↓ Streptococcus, Neisseria | ↑ Uremic toxins (urea, indoxyl sulfate), Oxidative stress, IL-18, IL-6, TNF-α, Disrupted lipid metabolism | Uremic milieu alters oral ecology → systemic inflammation and endothelial damage → cognitive decline via kidney–brain–oral inflammatory axis |
| Autoimmune/Inflammatory Conditions (e.g., Spondyloarthritis) | ↓ Streptococcus spp. (Bacilli)  ↑Veillonellaceae, Proteobacteria (Campylobacter concisus, Brucella spp.) | ↑ TNF-α, IL-17, IL-23; Dysregulated Th17 responses | Immune-mediated oral dysbiosis may amplify systemic inflammation and microglial activation, accelerating neurodegenerative changes |
